# Contribution of *KRAS* mutations and c.2369C > T (p.T790M) *EGFR* to acquired resistance to EGFR-TKIs in *EGFR* mutant NSCLC: a study on circulating tumor DNA

**DOI:** 10.18632/oncotarget.6957

**Published:** 2016-01-20

**Authors:** Marzia Del Re, Marcello Tiseo, Paola Bordi, Armida D'Incecco, Andrea Camerini, Iacopo Petrini, Maurizio Lucchesi, Alessandro Inno, Daniele Spada, Enrico Vasile, Valentina Citi, Giorgio Malpeli, Enrica Testa, Stefania Gori, Alfredo Falcone, Domenico Amoroso, Antonio Chella, Federico Cappuzzo, Andrea Ardizzoni, Aldo Scarpa, Romano Danesi

**Affiliations:** ^1^ Department of Clinical and Experimental Medicine, University of Pisa, Pisa, Italy; ^2^ Medical Oncology Unit, Azienda Ospedaliero-Universitaria, Parma, Italy; ^3^ Medical Oncology Unit, AUSL6, Istituto Toscano Tumori, Livorno, Italy; ^4^ Medical Oncology Unit, AUSL12, Istituto Toscano Tumori, Lido di Camaiore, Italy; ^5^ Department of Translational Research and New Technologies in Medicine and Surgery, University of Pisa, Pisa, Italy; ^6^ Medical Oncology Unit 2, Azienda Ospedaliero-Universitaria, Pisa, Italy; ^7^ Medical Oncology Unit, Ospedale Sacro Cuore, Negrar, Italy; ^8^ Medical Oncolgy Unit, Ospedale Santa Maria della Misericordia, Urbino, Italy; ^9^ ARC-NET Research Centre and Department of Pathology and Diagnostics, Azienda Ospedaliero-Universitaria, Verona, Italy; ^10^ Lung Diseases Unit, Azienda Ospedaliero-Universitaria, Pisa, Italy

**Keywords:** cell-free circulating tumor DNA, KRAS, EGFR, activating mutation, tyrosine kinase inhibitors

## Abstract

**Introduction:**

*KRAS* oncogene mutations (^MUT^*KRAS*) drive resistance to EGFR inhibition by providing alternative signaling as demonstrated in colo-rectal cancer. In non-small cell lung cancer (NSCLC), the efficacy of treatment with EGFR tyrosine kinase inhibitors (EGFR-TKIs) depends on activating *EGFR* mutations (^MUT^*EGFR*). However, inhibition of *EGFR* may select resistant cells displaying alternative signaling, i.e., *KRAS*, or restoration of EGFR activity due to additional ^MUT^*EGFR*, i.e., the c.2369C > *T* (^p.T790M^*EGFR*).

**Aim:**

The aim of this study was to investigate the appearance of ^MUT^*KRAS* during EGFR-TKI treatment and their contribution to drug resistance.

**Methods:**

This study used cell-free circulating tumor DNA (cftDNA) to evaluate the appearance of codon 12 ^MUT^*KRAS* and ^p.T790M^*EGFR* mutations in 33 advanced NSCLC patients progressing after an EGFR-TKI.

**Results:**

^p.T790M^*EGFR* was detected in 11 (33.3%) patients, ^MUT^*KRAS* at codon 12 in 3 (9.1%) while both ^p.T790M^*EGFR* and ^MUT^*KRAS* codon 12 were found in 13 (39.4%) patients. Six patients (18.2%) were *KRAS* wild-type (^WT^*KRAS*) and negative for ^p.T790M^*EGFR*. In 8 subjects paired tumor re-biopsy/plasma samples were available; the percent concordance of tissue/plasma was 62.5% for ^p.T790M^*EGFR* and 37.5% for ^MUT^*KRAS*. The analysis of time to progression (TTP) and overall survival (OS) in ^WT^*KRAS* vs. ^MUT^*KRAS* were not statistically different, even if there was a better survival with ^WT^*KRAS* vs. ^MUT^*KRAS*, i.e., TTP 14.4 vs. 11.4 months (*p* = 0.97) and OS 40.2 vs. 35.0 months (*p* = 0.56), respectively.

**Conclusions:**

^MUT^*KRAS* could be an additional mechanism of escape from EGFR-TKI inhibition and cftDNA is a feasible approach to monitor the molecular development of drug resistance.

## INTRODUCTION

Activating mutations of epidermal growth factor receptor (^MUT^*EGFR*) predict sensitivity to tyrosine kinase inhibitors (TKI) in non-small cell lung cancer (NSCLC). Despite a very high response rate (about 70%) to first-line treatment with the EGFR-TKIs (erlotinib, gefitinib or afatinib) in ^MUT^*EGFR* NSCLC, tumors invariably progress after a median of 9–13 months from the beginning of treatment [1–3].

The understanding of the molecular basis of acquired resistance to TKI [4] and its application to treatment monitoring may improve treatment management by discontinuing ineffective treatments and directing towards most appropriate second line options before clinical progression may occur. Indeed, EGFR signaling is maintained in most cases that develop secondary resistance [5] suggesting that additional molecular mechanisms can bypass EGFR-TKI inhibition reactivating the signaling pathway. Several mechanisms of acquired resistance to EGFR-TKI have been described after progression, including c.2369C > T (p.T790M) *EGFR* gatekeeper mutation (^p.T790M^*EGFR*, ~50% of patients) [6], *MET* (5–15%) [7] or *HER2* (12%) [8] amplifications, *PIK3CA* (4.1%) [9] or *BRAF* (1%) [10] mutations or transformation into small cell histology (3%) [11].

NSCLC heterogeneity can drive the therapeutic decisions [12]; therefore, tissue availability is increasingly recognized as a crucial issue. Unfortunately, the location of the tumor and the risk of complications are serious limitations to re-biopsies in NSCLC [13]. Alternatively, the detection of somatic mutations in cell-free tumor DNA (cftDNA) released in plasma could be instrumental for a better understanding of the genetic modifications driven by the selective pressure of drug treatments [14].

Interestingly, approximately 15–25% of patients with NSCLC have *KRAS* mutations (^MUT^*KRAS*), resulting in constitutive activation of *KRAS* signaling pathways. ^MUT^*KRAS* is a negative predictor of benefit to anti-EGFR antibodies in colo-rectal cancer, while it seems to be a negative predictor of response to EGFR-TKIs in *EGFR* wild type (^WT^*EGFR*) NSCLC patients [15]. In a previous study on a large collection of NSCLC tissues from patients with acquired resistance to EGFR-TKI, *NRAS* or *KRAS* mutations were not demonstrated [10]. Despite these negative results, we employed a sensitive ddPCR-based platform to investigate the presence of ^MUT^*KRAS* alleles in plasma of patients resistant to EGFR-TKIs and we were able to demonstrate a potential role of ^MUT^*KRAS* in acquired resistance to EGFR-TKI, besides the ^p.T790M^*EGFR*. This finding reveals a potential new mechanism of resistance to EGFR-TKI and underscores the need of a periodic monitoring of somatic mutations of known oncogenes to deliver the best personalized treatment in a timely fashion.

## RESULTS

The clinical characteristics of patients are reported in Table 1. Of 33 patients, 20 (60.6%) were female and 13 (39.4%) male. Median age was 62 years (range 41 – 75); 32 patients were affected by a stage IV disease, while one was a stage IIIB NSCLC. The frequency of activating ^MUT^*EGFR* was as follows: 20 patients (60.6%) showed ^ex19del^*EGFR*, 10 patients (30.3%) ^p.L858R^*EGFR*, 2 patients (6.1%) ^p.L747P^*EGFR* and 1 patient presented ^ex19ins^*EGFR* (3%). As expected, the majority of them (66.7%) was never-smokers, while 9 (27.2%) and 2 (6.1%) patients were former- and current-smokers, respectively. Twenty-seven (81.8%) subjects received gefitinib and 6 (18.2%) erlotinib; the treatment was administered as first-line in 23 (69.7%) (including 2 as maintenance), second-line in 6 (18.2%) and third or further lines in 4 patients (12.1%). The majority of them (66.7%) presented partial response to TKI treatment and only 1 patient showed complete response (Table 1). Stable and progressive diseases were observed in 4 (12.1%) and 6 subjects (18.2%), respectively. Patients who have progressed on EGFR-TKI treatment, all receiving gefitinib, presented the following molecular profile in their primary tumors: ^p.L747P^*EGFR* and ^ex19del^*EGFR* (*n* = 1 each) and ^p.L858R^*EGFR* (*n* = 4). Median time to progression (TTP) was 13.6 months (95% Confidence Interval, CI, range 8.0 – 19.2 months) and median overall survival (OS) was 40.2 months (95% CI range 25.8–54.7 months) for the overall population.

**Table 1:** Characteristics of patients

		No. (%)
Age years (range)		62 (41 – 75)
Gender	male	13 (39.4%)
	female	20 (60.6%)
Smoking habit	smokers	2 (6.1%)
	never-smokers	22 (66.7%)
	former-smokers	9 (27.2%)
Stage	IIIB	1 (3%)
	IV	32 (97%)
EGFR activating mutation	ex19del	20 (60.6%)
	p.L858R	10 (30.3%)
	p.L747P	2 (6.1%)
	ex19ins	1 (3%)
Line of treatment	1	23 (69.7%)
	2	6 (18.2%)
	≥ 3	4 (12.1%)
TKI	gefitinib	27 (81.8%)
	erlotinib	6 (18.2%)
Response	CR	1 (3%)
	PR	22 (66.7%)
	SD	4 (12.1%)
	PD	6 (18.2%)
TTP months (95% CI)		13.6 (8.0 – 19.2)
OS months (95% CI)		40.2 (25.8 – 54.7)

Abbreviations: TKI: tyrosine kinase inhibitor; TTP: time to progression; OS: overall survival

**Figure 1:**
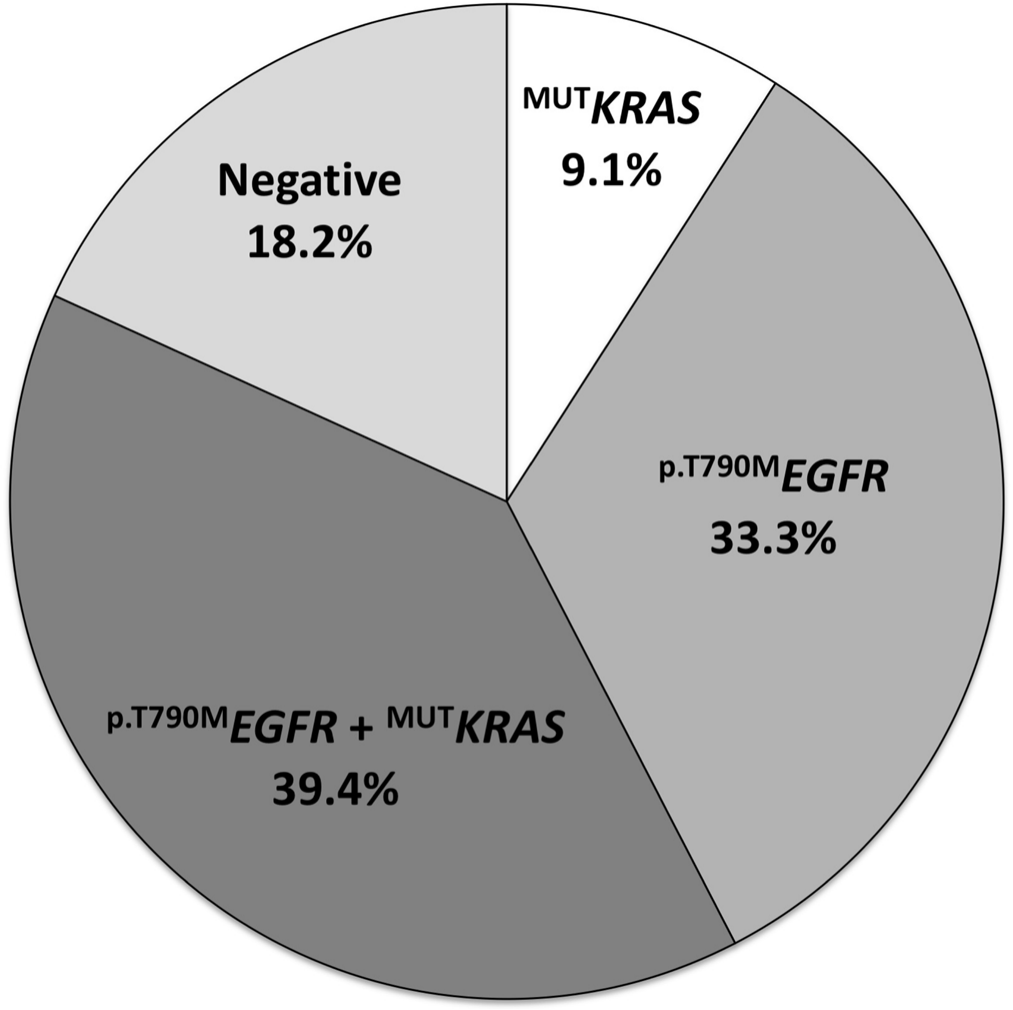
Occurrence of KRAS and p.T790M mutations in cftDNA of NSCLC patients treated with EGFR-TKI

The description of patients with activating ^MUT^*EGFR* in their primary tumors as well as the percentages of ^p.T790M^*EGFR* and ^MUT^*KRAS* alleles in cftDNA at the time of EGFR-TKI progression is reported in Table 2. In 16 patients (48.5%), a codon 12 ^MUT^*KRAS* was detected in cftDNA (Figure 1). In addition to this, the ^p.T790M^*EGFR* (c.2369C > T) second site mutation was present in the cftDNA of 24 patients (72.7%). Interestingly, 13 patients (39.4%) had both the ^MUT^*KRAS* and ^p.T790M^*EGFR*, while 3 (9.1%) and 11 (33.3%) subjects displayed only ^MUT^*KRAS* or ^p.T790M^*EGFR*, respectively (Figure 1). Six subjects displayed no mutations.

**Table 2:** Types of activating mutations of ^MUT^*EGFR* in primary tumor and % of ^p.T790M^*EGFR* and ^MUT^*KRAS* alleles in cftDNA. “-“ Indicates wild-type allele

Sample	Activating ^MUT^*EGFR*	^p.T790M^*EGFR %*	^MUT^*KRAS %*
1	Ex19del	7%	2%
**2**	p.L858R	11%	**-**
3	Ex19del	1%	1%
4	Ex19del	3%	2%
5	Ex19del	-	1%
6	Ex19del	-	1.5%
7	Ex19del	1.5%	1%
8	p.L858R	1%	-
9	Ex19del	-	-
10	Ex19del	-	-
11	p.L858R	-	-
12	p.L858R	5%	-
13	Ex19del	-	-
14	p.L747P	2%	-
15	p.L858R	-	-
16	Ex19del	1%	-
17	Ex19del	1%	2%
18	Ex19del	16%	1%
19	p.L858R	23%	1%
20	Ex19del	96%	98%
21	Ex19del	-	-
22	Ex19ins	-	1%
23	Ex19del	33%	2%
24	p.L747P	1%	1%
25	p.L858R	5%	2%
26	p.L858R	3%	1%
27	Ex19del	9%	3%
28	p.L858R	14%	-
29	Ex19del	28%	-
30	p.L858R	7%	-
31	Ex19del	39%	-
32	Ex19del	6%	-
33	Ex19del	43%	-

The association between smoking habit and the occurrence of ^MUT^*KRAS* in cftDNA was investigated. Regarding the 11 patients with smoking history, 2 (18.2%) presented ^MUT^*KRAS* and 9 (81.8%) were wild-type (^WT^*KRAS*). On the contrary, of 22 non-smoking subjects, 14 (63.3%) were ^MUT^*KRAS* and 8 (36.4%) ^WT^*KRAS*. Fisher's extact test revealed that non-smoking habit and ^MUT^*KRAS* were significantly associated (*p* = 0.026).

In 8 patients, paired re-biopsies and cftDNA were available. The 8 re-biopsies were performed in a different tumor site with respect to the initial diagnosis, the choice being dependent on several factors, i.e., anatomical accessibility, new or progressing lesions. The analysis of re-biopsies by standard methods and ddPCR demonstrated ^p.T790M^*EGFR* in 4 (standard) vs. 2 (ddPCR) samples and ^MUT^*KRAS* in none (standard) vs. 3 (ddPCR) specimens. ^p.T790M^EGFR and ^MUT^*KRAS* were detected in 7 and 5 cftDNA specimens, respectively.

The analysis of *KRAS* status by ddPCR in the biopsies at diagnosis revealed the presence of ^MUT^*KRAS* in 2 patients, who were ^WT^*KRAS* by standard method. Therefore, the percentage of patients developing ^MUT^*KRAS* as a mechanism of acquired resistance is 42% (14 patients). In terms of TTP and OS, there was no difference between these patients and the others (*p* = 0.19 and *p* = 0.13, respectively).

The concordance between tissue of re-biopsies (standard methods) and plasma (ddPCR), calculated by combining positive and negative results, was 62.5% and 37.5% for ^p.T790M^*EGFR* and ^MUT^*KRAS*, respectively (Table 3). Moreover, three paired samples found positive for the ^p.T790M^*EGFR* on re-biopsies (standard methods) and on cftDNA (ddPCR), were negative by ddPCR on primary tissue (Table 3).

**Table 3:** Molecular analysis of re-biopsies and comparison with cftDNA

Patient n.	Primary tumor		Re-biopsy				cftDNA	
	^MUT^*KRAS* std	^MUT^*KRAS* ddPCR	^p.T790M^*EGFR* ddPCR	^MUT^*KRAS* ddPCR	^p.T790M^*EGFR* std	^MUT^*KRAS* std	^p.T790M^*EGFR* ddPCR	^MUT^*KRAS* ddPCR
**2**	-	NA	-	-	-	-	Mut	-
**4**	-	Mut	Mut	Mut	-	-	Mut	Mut
**5**	-	Mut	-	Mut	-	-	-	Mut
**19**	-	-	-	-	Mut	-	Mut	Mut
**20**	-	-	-	-	-	-	Mut	Mut
**23**	-	-	Mut	Mut	Mut	-	Mut	Mut
**28**	-	NA	-	-	Mut	-	Mut	-
**30**	-	NA	-	-	Mut	-	Mut	-

Mut: presence; “-“: absence of mutation; NA: sample not available; std: standard sequencing approach.

The analysis of survival data stratified according to *KRAS* status in cftDNA, showed that TTP and OS were not statistically different, despite a trend towards a better survival in ^WT^*KRAS* vs. ^MUT^*KRAS* patients, i.e., TTP 14.4 vs. 11.4 months (*p* = 0.97) and OS 40.2 vs. 35.0 months (*p* = 0.56), respectively.

Finally, samples were analysed also for codons 13 (p.G13D) and 61 ^MUT^*KRAS* and ^MUT^*NRAS*, and ^V600E^*BRAF* and all the samples were found to be wild-type. Unfortunately, due to sample restriction, cftDNA was insufficient to perform the analysis of PI3K/Akt mutations.

## DISCUSSION

The present study demonstrates the presence of ^MUT^*KRAS* in the cftDNA of a significant proportion of patients progressing after EGFR-TKI treatment. In addition to this, the present study provides evidence that sensitizing ^MUT^*EGFR* and ^MUT^*KRAS* can coexist after the selective pressure of EGFR-TKI treatment.

^p.T790M^*EGFR* determines acquired resistance by increasing the affinity of EGFR to ATP [16, 17]. ^p.T790M^*EGFR* has been described in re-biopsies of 50–63% of tumors progressing under EGFR-TKI treatment [4, 11] and in the cftDNA at a frequency similar to our study [18–20]. Because drugs active on ^p.T790M^*EGFR*, such as AZD9291 and rociletinib [21, 22], are under clinical study and will be available soon in the clinical practice, the identification of this molecular marker is of utmost clinical relevance.

In our study, ^p.T790M^*EGFR* was more frequent in ^L858R^*EGFR* patients than in ^ex19del^*EGFR* ones (80% vs. 67%); on the contrary, ^MUT^*KRAS* in cftDNA was detected in 55% of patients with ^ex19del^*EGFR* vs. 30% of patients with ^L858R^*EGFR*. To our knowledge, a mechanism of resistance depending on activating ^MUT^*EGFR* has not been previously reported; however, this cohort is too small to draw any conclusion.

^MUT^*EGFR* and ^MUT^*KRAS* are mutually exclusive in primary NSCLC and only anecdotal case reports described their coexistence [23, 24]. ^MUT^*KRAS* occurs in approximately 20% of NSCLC cases at diagnosis, more frequently in Caucasian population, adenocarcinomas, males and current smokers [25, 26]. About 90% of *KRAS* mutations occur in exon 2 (codon 12 and 13), while exon 3 (codon 61) is less frequently involved [26, 27]; in never-smokers with lung adenocarcinoma, ^MUT^*KRAS* is more frequently a transition (G to A) compared to transversion in current smokers [25]. Colo-rectal cancer cells with ^MUT^*KRAS* treated with anti-EGFR monoclonal antibodies are able to escape growth inhibition by several mechanisms, including ^MUT^*RAS* [28]. While the role of ^MUT^*KRAS* in primary resistance to EGFR-TKIs in molecularly unselected NSCLC is quite well established [29, 30], its development and role in acquired resistance to EGFR-TKIs in ^MUT^*EGFR* patients has not been explored in detail. In a previous work on a large collection of NSCLC tissues from patients with acquired EGFR-TKI resistance, ^MUT^*NRAS* or ^MUT^*KRAS* were not demonstrated [10]. However, comparison with the present results is not possible because detailed information were not provided neither on the timing of sampling with respect of development of TKI resistance nor on the type of tissue analysed. Therefore, we addressed this issue and a sensitive ddPCR-based platform was employed to investigate the presence of ^MUT^*KRAS* alleles besides the well-known ^p.T790M^*EGFR*. Due to its high sensitivity, ddPCR is able to identify small amounts of ^MUT^*KRAS* and many methodological issues need to be addressed prospectically, particularly the threshold level of both ^MUT^*KRAS* and ^p.T790M^*EGFR* to be considered clinically relevant. However, a mechanism of drug resistance does not necessarily reflect biologic aggressiveness and the lack of difference in OS between *KRAS* wild-type and mutated patients it is therefore not surprising. The numeric dimension of the cell clone bearing ^MUT^*KRAS* should be taken into consideration as well. Nevertheless, despite the low proportion of smokers in our cohort, the high prevalence of ^MUT^*KRAS* could support its role of as mechanism of acquired resistance.

Eight patients underwent re-biopsy after tumor progression during EGFR-TKI, allowing a comparison between tissue and cftDNA. The detection of mutations in cftDNA but not in re-biopsy, using both standard methods and ddPCR, could suggest the presence of heterogeneity within metastatic sites or the lower performance of ddPCR in the presence of paraffin. Nevertheless, the detection of mutations in both plasma and tissue by ddPCR, but not by standard methods, could be due to the higher sensitivity of ddPCR analysis. Two patients, initially diagnosed ^WT^*KRAS* by standard method, were re-analysed by ddPCR and were found ^MUT^*KRAS* in the primary biopsy, suggesting that the ^MUT^*KRAS* clone co-existed with activating ^MUT^*EGFR* since the beginning, as also demonstrated in previous reports [23, 24]. In these patients, ^MUT^*KRAS* cannot strictly be considered a mechanisms of resistance but it could be possible that EGFR-TKI treatment may have favored the expansion of ^MUT^*KRAS*-positive clones. However, conclusions cannot be drawn as pre-treatment cftDNA was not available.

Our observation is a pivotal evidence of the presence of ^MUT^*KRAS* in cftDNA of tumors with sensitizing ^MUT^*EGFR* resistant to EGFR-TKIs. A small percentage of our patients received EGFR-TKI as third line therapy and a new biopsy was not repeated at this time. It could be possible that ^MUT^*KRAS* appeared before the initiation of TKI as a mechanism of resistance to previuos therapy. This hypotesis is weakened by the evidence that patients given second or further lines of therapy showed TTP and OS similar to patients treated in first line, although a mechanism of resistance does not necessarily affect survival. The presence of ^MUT^*KRAS* has been recently reported using next generation sequencing analysis of tumor re-biopsies after progression under EGFR-TKI treatment [31], similarly to colo-rectal cancer treated with EGFR antibodies [32, 33]. It remains to be determined if the presence of ^p.T790M^*EGFR* and ^MUT^*KRAS* coexist in the same tumor cell or arise in different subclones.

Targeting ^MUT^KRAS proteins is still a challenge [34, 35]. Theoretically, combined treatment with KRAS and EGFR inhibitors can be administered to patients to prevent ^MUT^KRAS-dependent resistance or restore sensitivity to EGFR-TKIs, as recently demonstrated co-targeting EGFR and MEK [36]. To date, ^p.T790M^*EGFR* remains the most important predictor of efficacy of third generation EGFR-TKIs. Moreover, it was found that the coexistence of both activating ^MUT^*EGFR* and ^MUT^*KRAS* was not necessarily a negative predictor for EGFR-TKI therapy [23]. With these evidences in mind, all patients with ^p.T790M^*EGFR* should receive third generation EGFR-TKI, even in the presence of ^MUT^*KRAS*. Theoretically, it is possible that ^MUT^*KRAS* identify a less responsive subgroup of patients but this hypothesis should be validated by monitoring patients prospectically during second-line therapy. Beside ^p.T790M^*EGFR* and ^MUT^*KRAS*, other mechanisms of acquired resistance not evaluated in our study have been described in tumor re-biopsies after EGFR-TKI progression, including actionable mutations of *MET* [7], *HER2* [8], *PIK3CA* [9] or transformation into small cell histology (3%) [11].

In conclusion, despite the small number of patients involved, the retrospective analysis and the low rate of re-biopsies, our results confirm the importance of cftDNA analysis for the monitoring of secondary mutations associated with EGFR-TKI resistance in NSCLC and underline the role of a highly sensitive approach, i.e., ddPCR, for the detection of low-level mutations. The clinical relevance of these findings, expecially for what concerns ^MUT^*KRAS*, needs to be evaluated prospectively. These observations open new perspectives in the molecular mechanisms of acquired resistance, indicating a possible role of ^MUT^*KRAS* in tumor escape from pharmacologic treatment. The effect of ^MUT^*KRAS* in NSCLC with activating ^MUT^*EGFR* needs to be further elucidated at the molecular level and encourages the development of inhibitors ^MUT^*KRAS* for optimal treatment of patients.

## MATERIALS AND METHODS

### Study population

A total of 33 NSCLC patients with activating ^MUT^*EGFR* (exon 19 deletion [^ex19del^*EGFR*], exon 21 c.2573T > G [^p.L858R^*EGFR*] or exon 19 c.2240T > C [^p.L747P^*EGFR*]), receiving EGFR-TKI (gefitinib or erlotinib) as per approved indication were included in this study. The analysis of EGFR mutations in primary tumors was performed by standard diagnostic procedures in use in each centre participating to this study (i.e., EGFR TKI response^®^, Diatech, Jesi, Italy; Therascreen^®^, Qiagen, Valencia, CA, USA). ^MUT^*KRAS* were not examined at the time of diagnosis because mutually exclusive with activating ^MUT^*EGFR*. Plasma and/or re-biopsy samples were taken at the time of disease progression. The analysis of ^MUT^*KRAS* mutations and *EGFR* c.2369C > T (^p.T790M^*EGFR*) in plasma was not part of standard clinical management and the study was submitted and approved by the Ethics Committee of Pisa University Hospital and conducted in accordance to the principles of the Declaration of Helsinki; all patients gave their signed informed consent before blood collection and cftDNA analysis.

### Plasma collection and cftDNA extraction

Six ml of blood were collected in EDTA and centrifuged at 4°C for 10 min at 3000 rpm within two hours after blood drawing. Plasma samples were stored at −80°C until analysis. cftDNA was extracted using a QIAmp Circulating nucleic acid Kit (Qiagen^®^, Valencia, CA, USA) from 1 to 3 ml of plasma following the manufacturer's protocol and the DNA was eluted in 100 μl of buffer.

### Analysis of cftDNA

The investigational part of this study included the assessment of codons 12, 13 (p.G13D) and 61 ^MUT^*KRAS* and ^MUT^*NRAS*, ^p.T790M^*EGFR*, ^V600E^*BRAF* in cftDNA. Other mutations potentially associated with EGFR-TKI resistance were not examined because of the limited amount of cftDNA available. The analysis of cftDNA was performed by digital droplet PCR (ddPCR, BioRad^®^, Hercules, CA, USA) and ddPCR Mutation Assay (BioRad^®^). The analytic procedure was unable to discriminate the nature of the ^MUT^*KRAS* because the analysis was performed with a ddPCR KRAS Multiplex assay.

PCR reactions were assembled into individual wells of a single-use injection molded cartridge, according to the following protocol: 20 ng of template DNA (4 μl), 1 μl of 20X target primer/probe assay (FAM), 1 μl of 20X wild type primer/probe assay (HEX), 10 μl of 2X ddPCR Super Mix and 4 μl of DNAse/RNAse-free water up to a total volume of 20 μl. Droplet generation oil (70 μl) was then loaded and the cartridge was placed into the droplet generator. Using vacuum, sample and oil were mixed, generating mono-disperse droplets. Thereafter, 40 μl of packed droplets were transferred into a 96-well PCR plate for thermal cycling amplification. The protocol was standardized for all mutations to the following conditions: 95°C × 10 min, 94°C × 30 s and 55°C × 60 s (35 cycles), 98°C × 10 min, and 4°C hold. The droplet reader (BioRad^®^) was used for fluorescence signal quantification. The concordance between ^MUT^*KRAS* and ^p.T790M^*EGFR* was assessed on pairwise cftDNA and tissue DNA of 8 patients who underwent re-biopsy for diagnostic purposes. DNA was extracted from formalin-fixed paraffin-embedded biopsies using the QIAmp DNA Mini Kit (Qiagen^®^) and analyzed using conventional diagnostics as reported above. As a positive control for ^MUT^*KRAS*, the cftDNA from 30 patients with known ^MUT^*KRAS* pancreatic cancer was used, while the DNA extracted from plasma of 43 healthy blood donors was employed as negative control for ^MUT^*KRAS* and ^p.T790M^*EGFR*.

### Data analysis

TTP and OS were calculated following standard procedures and survival curves were generated by the SPSS statistical software (version 16).
